# Predictive value of the stone-free rate after percutaneous nephrolithotomy based on multiple machine learning models

**DOI:** 10.3389/fmed.2025.1559613

**Published:** 2025-08-19

**Authors:** Zhao Rong Liu, Zhan Jiang Yu, Jie Zhou, Jian Biao Huang

**Affiliations:** ^1^Jiangxi Cancer Hospital & Institute, Jiangxi Clinical Research Center for Cancer, The Second Affiliated Hospital of Nanchang Medical College, Nanchang, China; ^2^Department of Urology, Yudu County People’s Hospital, Yudu, China; ^3^The Second Affiliated Hospital, Jiangxi Medical College, Nanchang University, Nanchang, China

**Keywords:** urinary tract stones, percutaneous nephrolithotomy, machine learning, stone-free rate, predict

## Abstract

**Purpose:**

This study aimed to develop three types of machine learning (ML) models based on gradient boosting decision tree (GBDT), random forest (RF), and extreme gradient boosting (XGBoost) to explore their predictive value for the stone-free rate after percutaneous nephrolithotomy (PCNL).

**Patients and methods:**

A retrospective analysis was conducted on 160 patients who underwent PCNL. The patients were randomly divided into a training set and a test set in a 7:3 ratio. Clinical data were collected, and univariate analysis was performed to identify important data significantly associated with the stone-free rate after PCNL. Three ML models (GBDT, RF, and XGBoost) were developed using the training set. The predictive performance of these models was evaluated using the area under the curve (AUC) of the receiver operating characteristic (ROC) on the test set, confusion matrix, specificity, sensitivity, accuracy, and F1 score. For the top-performing prediction model, the study further employed the SHapley Additive exPlanations (SHAP) method to enhance model interpretability by elucidating the contribution of individual features to the prediction outcomes and ranking the relative importance of the predictive data. Finally, the clinical utility of the model was assessed through decision curve analysis (DCA), which quantified the net clinical benefit of applying the model across various risk thresholds.

**Results:**

Postoperative statistics indicated a stone-free rate of 70.6% (*n* = 113) among the patients. The data significantly associated with the absence of residual stones included the number of stones, stone diameter, stone CT value, history of previous stone surgery, stone location, and stone shape (*p* < 0.05). All three models demonstrated strong predictive effects in the validation set, with the GBDT model showing superior performance [AUC: 0.836 (95% CI: 0.785–0.873); accuracy: 0.854; sensitivity: 0.853; specificity: 0.857] compared to the XGBoost [AUC: 0.830 (95% CI: 0.792–0.868); accuracy: 0.771; sensitivity: 0.824; specificity: 0.643] and RF models [AUC: 0.803 (95% CI: 0.763–0.837); accuracy: 0.792; sensitivity: 0.824; specificity: 0.714]. The F1 scores for the GBDT, RF, and XGBoost models were 0.892, 0.836, and 0.849, respectively. The DCA decision curve analysis confirmed that the GBDT model offers a favorable net clinical benefit. In addition, the SHAP analysis identified the number of stones and the stone CT value as the most critical features influencing the model’s predictions, contributing significantly to its overall predictive performance.

**Conclusion:**

The prediction models developed based on three machine learning algorithms can accurately predict the stone-free rate after PCNL in patients with urinary tract stones. Among these, the GBDT model can effectively identify patients who are most likely to achieve successful outcomes from PCNL based on demographic and stone characteristics, thereby assisting in clinical treatment decision-making.

## Introduction

As one of the most common conditions in urology, the global incidence of urinary tract stones ranges from 5 to 10%, with a higher proportion observed in North America and Europe (1). The medical costs associated with urinary tract stones are substantial due to emergency medical treatment, surgical interventions, and other related healthcare expenses. Some studies estimate that the annual direct medical costs of urinary tract stones in China may exceed 10 billion yuan (approximately US$1.5 billion) (2, 3). Percutaneous nephrolithotomy (PCNL) is characterized by minimal trauma, rapid recovery, a high stone clearance rate, and fewer complications compared to open surgery, making it widely utilized in clinical practice. It has gradually become the first-line surgical method for complex stones and those with a diameter greater than 2 cm (4, 5). However, factors such as the location and size of the stones may impact the surgical outcome, potentially leading to PCNL failure and resulting in incomplete stone removal, bleeding, or infection. Even with optimal surgical techniques, these situations can occasionally occur (6). Residual stones not only adversely affect patients’ quality of life but also pose a risk of requiring secondary surgery, significantly impacting the overall recovery following urinary stone surgery (7). Consequently, accurately identifying the potential for residual stones after PCNL and understanding the postoperative stone-free rate are of utmost importance.

Machine learning, a branch of artificial intelligence, employs algorithms and statistical models to enable computer systems to automatically analyze training data, recognize patterns, and make new decisions without explicit programming. In machine learning, a model is developed by learning from training data and can predict or classify new data (8, 9). Machine learning (ML) models can assist physicians in predicting diagnoses, grading, and prognoses of urological diseases by analyzing patients’ clinical data, imaging results, and laboratory indicators (10, 11). A machine learning model that integrates radiomics and clinical data can effectively identify urinary tract infection stones *in vivo*, potentially optimizing the management of urolithiasis and improving patient outcomes (12). Moreover, a model based on machine learning algorithms has proven to be quite accurate in predicting postoperative bleeding after PCNL in the contralateral decubitus position, aiding urologists in making informed treatment decisions (13).

This study developed three new machine learning prediction models for stone-free rates after PCNL, aiming to identify the most suitable patients for the procedure preoperatively, thereby enhancing perioperative management and preventing the occurrence of postoperative stone residues.

## Patients and methods

### Patients

A total of 160 eligible patients who underwent PCNL at the Second Affiliated Hospital of Nanchang University from April 2021 to November 2022 were included in this retrospective analysis. The inclusion criteria were as follows: (1) patients diagnosed with kidney stones and/or upper ureteral stones by a CT scan within 1 month prior to the procedure; (2) patients who received complete PCNL surgical treatment; and (3) availability of complete clinical data. The exclusion criteria included the following: (1) presence of malignant tumors in the urinary system on the ipsilateral side; (2) severe liver and renal dysfunction or systemic coagulation disorders; and (3) congenital malformations or severe atrophy of the ipsilateral kidney. All surgeries were performed using a standard single-channel technique. The stone-free rate was defined as the absence of residual stones or the presence of remaining stones measuring less than 4 mm, as assessed by a plain X-ray examination of the urinary tract approximately 1 week after surgery. All patients were randomly divided into a training set (*n* = 112) and a test set (*n* = 48) in a ratio of 7:3, using the random seed method in the R language.

### Data collection and processing

By querying the hospital’s medical record system, we obtained the clinical information of all patients, which included the following: (1) general conditions: age, sex, BMI, hypertension, and diabetes; (2) preoperative conditions: preoperative stent placement or nephrostomy, the extent of preoperative infection and hydronephrosis (defined by preoperative B-ultrasound as mild if the separation of the renal collecting system was 2–3 cm, moderate if 3–4 cm, and severe if greater than 4 cm), surgeon experience (surgeons who have performed more than 100 PCNL procedures were considered experienced, whereas those with fewer than 100 procedures were regarded as less experienced), and history of stone surgery (including PCNL and non-PCNL procedures such as ureteroscopic lithotripsy and open stone surgery); and (3) stone characteristics: affected sides, number of stones, stone location, maximum stone diameter (the diameter of the largest single stone), stone CT value [average CT value representing stone density, measured in Hounsfield units (HU)], and stone shape. Important data were identified through univariate analysis.

### Model building and evaluation

Gradient boosting decision tree (GBDT) is a process that combines multiple weak classifiers offline to create a strong classifier. This algorithm significantly enhances the flexibility and convenience of making predictions based on decision trees. Random forest (RF) is an ensemble learning method that relies on numerous decision trees, with the output category determined by the majority vote from the individual trees. Extreme gradient boosting (XGBoost) efficiently implements GBDT while incorporating numerous improvements in both algorithms and engineering practices. The screened clinical data mentioned above were used to construct the machine learning models. Three types of machine learning algorithms were trained using a binary classification approach. The training set (70%) was utilized to train the models, while the validation set (30%) was employed to evaluate the models’ predictive performance. By calculating the prediction accuracy, specificity, sensitivity, F1 score, confusion matrix, and area under the curve (AUC), we compared the predictive values of the GBDT, RF, and XGBoost models. The SHapley Additive exPlanations (SHAP) method was used to evaluate the contribution of individual features within the optimal predictive model, while the clinical net benefit was quantified through decision curve analysis (DCA).

### Surgical methods

All patients were placed in the standard lithotomy position before surgery to examine the bladder and ureter on the surgical side. Subsequently, a 5F ureteral catheter was inserted into the ureter on the surgical side, advanced 25–28 cm, secured in place, and connected to an infusion device to induce artificial hydronephrosis; the duration of artificial hydronephrosis induction varied depending on the severity of the patient’s hydronephrosis. Following repositioning to the prone position and under ultrasound guidance, the renal calyx was punctured, with the approach determined by preoperative imaging. The supracostal upper pole approach was preferred for pelvic or large stones, while subcostal or lower pole punctures were employed for isolated lower or mid-pole calyx stones. Once the needle core was withdrawn and urine was observed flowing out, a renal puncture guidewire was inserted through the puncture needle sheath. Guided by the wire, an F10 fascia dilator was used to gradually expand the tract to F24 in 2F increments, followed by the insertion of an F24 tearable sheath, which possesses a tearable feature, allowing it to maintain the sinus tract while being flexible enough for appropriate deformation. Subsequently, an L-type nephroscope (24Fr) was introduced to confirm the location of the stones. The stones were then fragmented using a holmium laser, with smaller fragments irrigated out and larger fragments removed using grasping forceps. All procedures adhered to the standard PCNL protocol and were successfully completed through a single puncture channel. An F6 double-J stent was placed postoperatively and maintained for approximately 4 weeks.

### Statistical analysis

Data analysis and processing were performed using SPSS version 27.0, R language software (version 4.3.1),^1^ and Python language software.^2^ All measurement data are expressed as mean ± standard deviation, while categorical data are presented as numbers (%). A single-factor analysis was conducted to compare differences in clinical factors. The *t*-test or Mann–Whitney *U*-test was employed for measurement data, and the chi-squared test or Fisher’s exact test was used for categorical data. A *p*-value of <0.05 was considered statistically significant. Lasso regression was performed using the glmnet package in R or the scikit-learn package in Python. The matplotlib package in Python was utilized to generate the receiver operating characteristic (ROC) curve.

## Results

### Patients

According to postoperative review statistics, among the 160 patients, 47 had residual stones, while 113 had completely cleared stones, resulting in a stone-free rate of 70.6%. The number of stones, stone diameter, stone CT value, history of previous stone surgery, stone location, and stone shape were found to be significantly associated with postoperative stone clearance (*p* < 0.05) (see Table 1).

**Table 1:** Basic characteristics and difference analysis.

Data	Total (*n* = 160)	Non-SF (*n* = 47)	SF (*n* = 113)	Statistic	*p*
Age, year, mean ± SD	54.35 ± 12.55	54.94 ± 11.31	54.11 ± 13.07	*t* = 0.38	0.704
BMI, mean ± SD	23.49 ± 3.47	23.52 ± 3.84	23.48 ± 3.33	*t* = 0.06	0.949
Stones number, M (Q₁, Q₃)	3 (2, 4)	5 (4, 6)	3 (1, 4)	*Z* = −6.77	<0.001
Stone diameter, cm, M (Q₁, Q₃)	2.0 (1.4, 2.6)	2.6 (2.0, 3.5)	1.7 (1.3, 2.3)	*Z* = −5.16	<0.001
Stone CT value, Hu, M (Q_1_, Q_3_)	1,140 (983, 1,280)	1,240 (1,070, 1,383)	1,080 (950, 1,210)	Z = −3.78	<0.001
Sex, *n* (%)				*χ*^2^ = 0.02	0.893
Male	100 (62.50)	29 (61.70)	71 (62.83)		
Female	60 (37.50)	18 (38.30)	42 (37.17)		
Pre-stenting/nephrostomy, *n* (%)				*χ*^2^ = 1.06	0.303
No	150 (93.75)	46 (97.87)	104 (92.04)		
Yes	10 (6.25)	1 (2.13)	9 (7.96)		
History of stone surgery, *n* (%)				*χ*^2^ = 6.04	0.049
No	122 (76.25)	30 (63.83)	92 (81.42)		
PCNL	16 (10.00)	8 (17.02)	8 (7.08)		
Non-PCNL surgery	22 (13.75)	9 (19.15)	13 (11.50)		
Pre-infection, *n* (%)				*χ*^2^ = 2.83	0.419
−	17 (10.62)	3 (6.38)	14 (12.39)		
+	84 (52.50)	24 (51.06)	60 (53.10)		
++	29 (18.12)	8 (17.02)	21 (18.58)		
+++	30 (18.75)	12 (25.53)	18 (15.93)		
Hypertension, *n* (%)				*χ*^2^ = 1.43	0.232
No	116 (72.50)	31 (65.96)	85 (75.22)		
Yes	44 (27.50)	16 (34.04)	28 (24.78)		
Diabetes, *n* (%)				*χ*^2^ = 0.00	1.000
No	148 (92.50)	43 (91.49)	105 (92.92)		
Yes	12 (7.50)	4 (8.51)	8 (7.08)		
Stone laterality, *n* (%)				*χ*^2^ = 0.02	0.877
Left	73 (45.62)	21 (44.68)	52 (46.02)		
Right	87 (54.38)	26 (55.32)	61 (53.98)		
Stone location, *n* (%)				*χ*^2^ = 7.48	0.024
Kidney	26 (16.25)	2 (4.26)	24 (21.24)		
Ureter	97 (60.62)	31 (65.96)	66 (58.41)		
Kidneys and ureters	37 (23.12)	14 (29.79)	23 (20.35)		
Hydronephrosis, *n* (%)				*χ*^2^ = 3.31	0.346
No	13 (8.12)	1 (2.13)	12 (10.62)		
Mild	81 (50.62)	26 (55.32)	55 (48.67)		
Moderate	35 (21.88)	11 (23.40)	24 (21.24)		
Severe	31 (19.38)	9 (19.15)	22 (19.47)		
Experience of operator, *n* (%)				*χ*^2^ = 2.44	0.118
PCNL <100	32 (20.00)	13 (27.66)	19 (16.81)		
PCNL >100	128 (80.00)	34 (72.34)	94 (83.19)		
Stone shape, *n* (%)				*χ*^2^ = 21.87	<0.001
Non-staghorn stones	96 (60.00)	15 (31.91)	81 (71.68)		
Staghorn stones	64 (40.00)	32 (68.09)	32 (28.32)		

BMI, body mass index; non-PCNL surgery: ureteroscopic lithotripsy, open stone surgery, and extracorporeal shock wave lithotripsy; pre-infection: + mild infection, ++ moderate infection, and +++ severe infection.

### Machine learning model building

We randomly assigned 70% of the patients to the training set and the remaining 30% to the test set. The stone-free rate was approximately the same in both the training set (70.5%) and the test set (70.8%). The machine learning algorithms were employed to construct predictive models based on the clinical data described above. Utilizing the Python scikit-learn framework, multiple decision trees were constructed, and their prediction results were combined. The model parameters were updated, and the objective function was optimized to enhance the accuracy of the model. This included utilizing second-order derivative information to optimize the loss function, ultimately achieving binary classification predictions of the stone-free rate post-PCNL.

### Model evaluation and comparison

**Figure 1:**
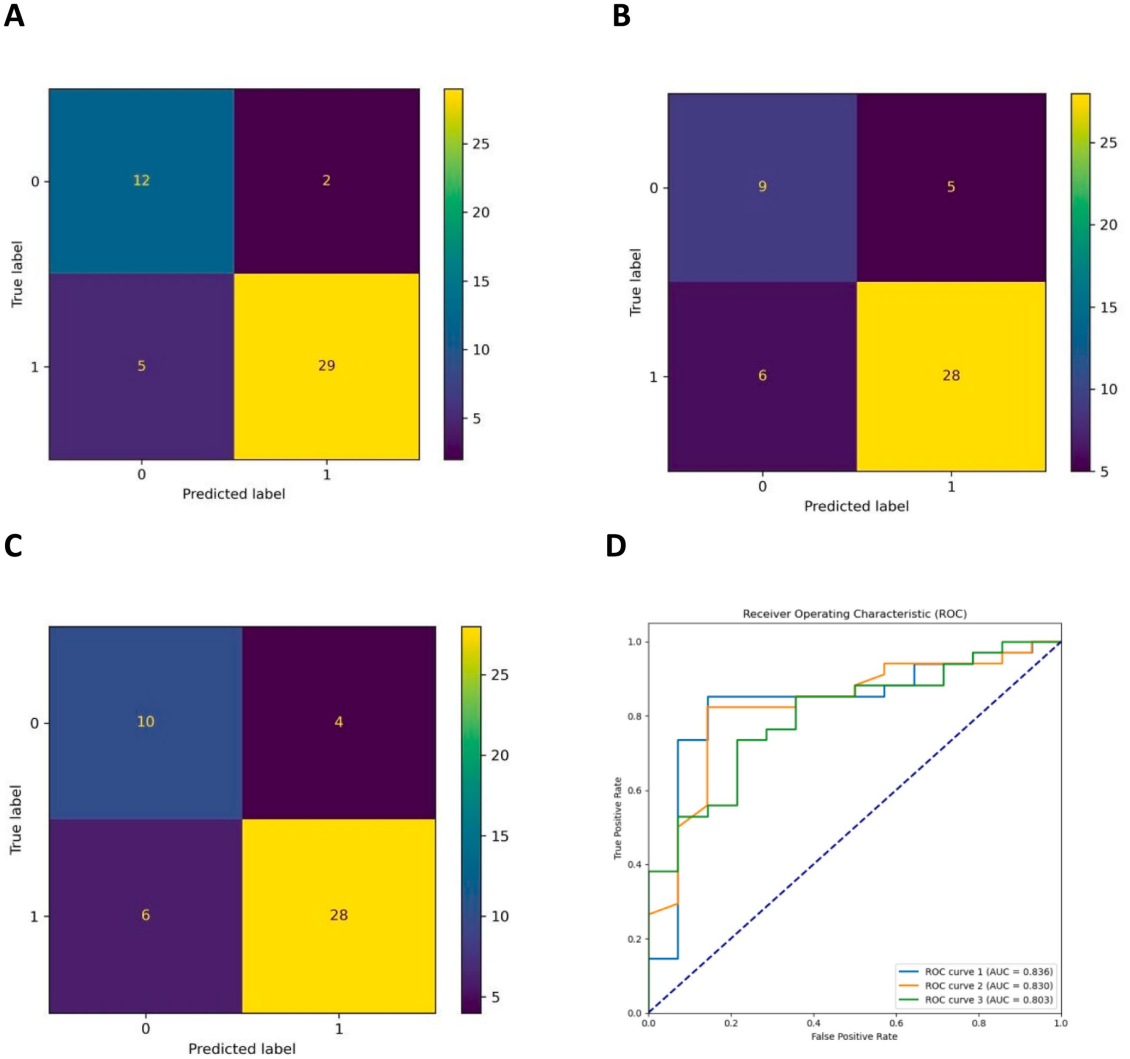
**(A–C)** Are the confusion matrix diagrams of the GBDT, XGBoost, and RF models, respectively, and **(D)** is the ROC curve of the three models (ROC curves 1, 2, and 3 are the GBDT, RF, and XGBoost models, respectively).

**Figure 2:**
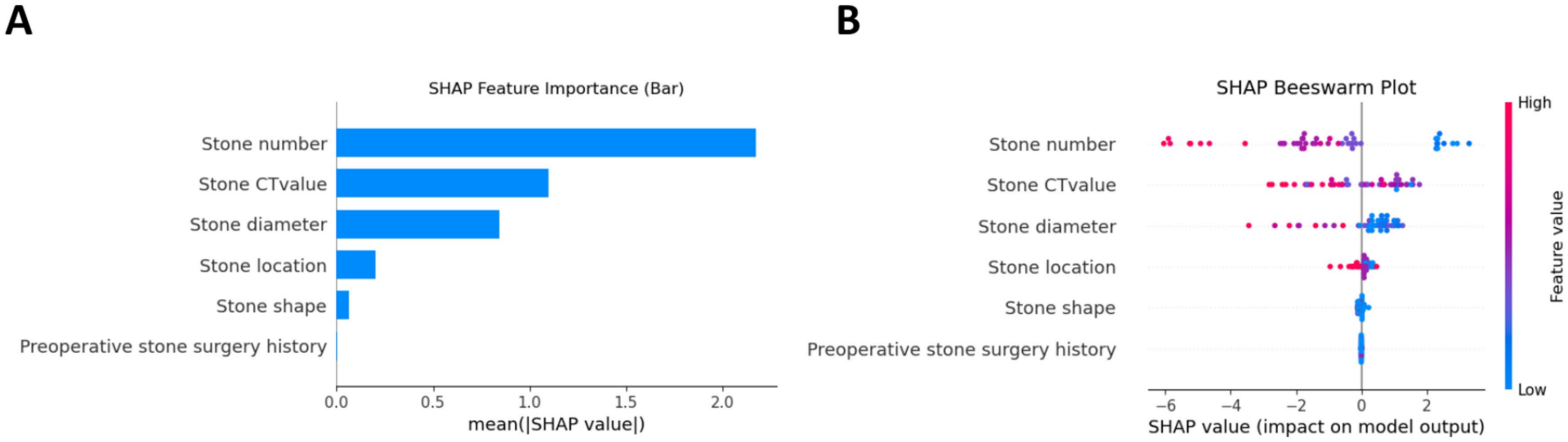
Interpretability analysis of the GBDT model using the SHAP method. **(A)** Feature importance ranking by mean absolute SHAP values. **(B)** SHAP summary plot demonstrating individual feature contributions, where each point represents a patient and color intensity corresponds to feature value magnitude and direction of prediction impact.

**Figure 3:**
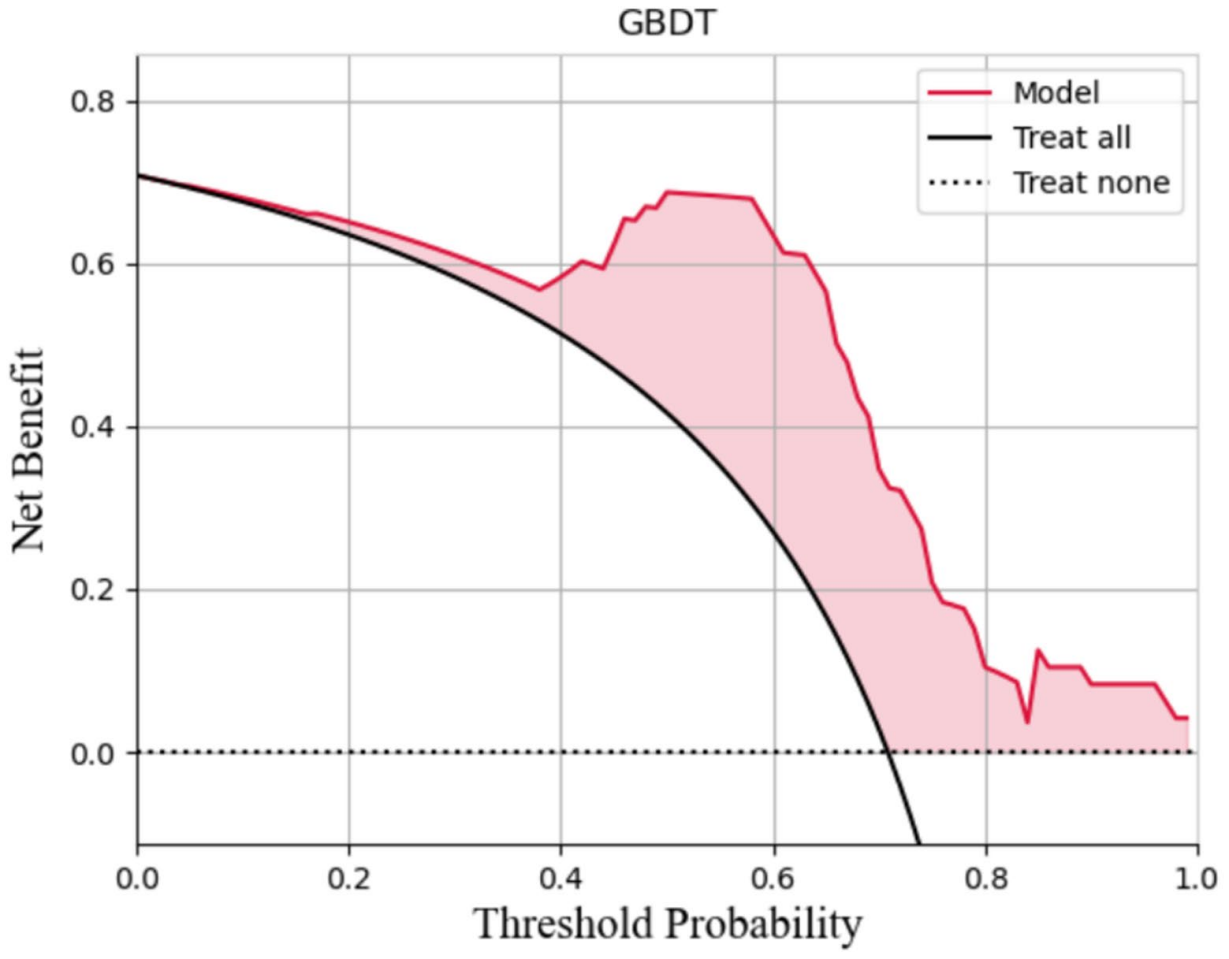
The DCA curve of the GBDT model.

The AUCs for the test sets of the GBDT, RF, and XGBoost models were 0.836 (95% CI: 0.785–0.873), 0.803 (95% CI: 0.763–0.837), and 0.830 (95% CI: 0.792–0.868), respectively. The accuracy, specificity, sensitivity, and F1 score of the three models are presented in Table 2. The confusion matrix and ROC curve are depicted in Figure 1. The sensitivity values for the three machine learning models ranged from 0.824 to 0.853, specificity values ranged from 0.643 to 0.857, accuracy values ranged from 0.771 to 0.854, and F1 scores ranged from 0.836 to 0.892. This indicates that all three models demonstrate strong predictive capabilities. After a comprehensive comparison of these metrics, particularly the prediction accuracy, the GBDT model was selected as the final prediction model. To further elucidate the decision-making process of the optimal model, we performed DCA and employed the SHAP method to analyze the top-performing GBDT model, ranking the importance of the predictive data. The SHAP analysis indicated that the number of stones and the stone CT value were the two most influential features affecting the model’s predictions (Figure 2), with their significant contributions thoroughly validated by the SHAP values (Figure 2). The DCA curve demonstrates that the GBDT model offers substantial clinical benefits to patients (Figure 3).

**Table 2:** AUC, accuracy, sensitivity, specificity, and F1 values of the different models in the test set.

Models	Accuracy	Sensitivity	Specificity	F1 score	AUC	95% CI
GBDT	0.854	0.853	0.857	0.892	0.836	0.785–0.873
XGBoost	0.771	0.824	0.643	0.836	0.803	0.736–0.837
RF	0.792	0.824	0.714	0.849	0.830	0.792–0.868

## Discussion

With the continuous development and advancement of minimally invasive surgical techniques and endoscopic instruments, the surgical treatment of urinary tract stones has gradually evolved into a system primarily based on transurethral ureteroscopy and percutaneous nephrolithotomy (PCNL) (14, 15). PCNL, in particular, is widely favored due to its minimal trauma and rapid recovery. Compared to traditional open surgery, PCNL has significantly improved stone clearance rates, reduced postoperative complications, and shortened hospital stays. However, despite the maturation of PCNL technology, its effectiveness and safety across different patient groups still require further investigation (16, 17). Factors such as a patient’s anatomical characteristics, the size and nature of the stone, and the presence of comorbidities can all impact surgical outcomes. The removal of larger or more complex stones inevitably increases the risk of residual stones (18, 19). Therefore, when selecting treatment options, it is essential to thoroughly evaluate the individual characteristics of each patient and to develop a personalized treatment strategy.

Extensive previous research has successfully developed nomogram-based prediction models to accurately evaluate treatment outcomes following PCNL (20). With the increasing application of artificial intelligence approaches, particularly machine learning methods, in medical research, these advanced techniques have demonstrated remarkable advantages in predicting surgical outcomes. This advancement highlights the significant potential of machine learning-based models in predicting postoperative outcomes of PCNL, with particular focus on residual stones—a crucial clinical endpoint that greatly influences patient management (21–23). The application of machine learning in the diagnosis and treatment of urinary tract stones is emerging as a significant research focus in urology (24, 25). By analyzing extensive clinical data, machine learning can identify risk factors unique to different patients, predict the occurrence, progression, and treatment outcomes of stones, and assist in optimizing treatment plans (26–28). A study by Shee et al. (29) found that a machine learning model based on 24-h urine data could predict stone recurrence with moderate accuracy. In addition, the machine learning model developed by Zhang et al. (30), which utilized various laboratory test indicators and stone characteristics, was able to accurately predict the likelihood of systemic inflammatory response syndrome following PCNL, thereby aiding surgeons in their clinical decision-making. These automated data analysis models founded on machine learning not only enhance work efficiency but also reduce human error, providing clinicians with robust decision-making support (31, 32).

Machine learning models demonstrate theoretical potential for predicting stone-free rates in PCNL procedures, as they primarily analyze stone characteristics and clinical data (13, 33). While core predictive features (stone morphology, density, and anatomical parameters) are fundamentally important across different surgical approaches, it should be emphasized that our current model was exclusively developed and validated using prone-position PCNL data. Therefore, while these algorithms may conceptually maintain relevance for supine PCNL and miniaturized techniques, their actual clinical utility for these specific variants remains to be prospectively validated. This important limitation must be considered when interpreting the model’s generalizability across different PCNL approaches. Future studies specifically evaluating the algorithm’s performance in supine and mini-PCNL settings are needed to confirm its broader applicability.

This study utilized meaningful clinical data to develop prediction models based on GBDT, XGBoost, and RF algorithms. By calculating the accuracy, specificity, sensitivity, F1 score, ROC curve, and confusion matrix of the three models, we investigated the applicability of machine learning methods in preoperative predictions of stone-free rates after PCNL. Among these three models, the GBDT model demonstrated the highest AUC value and accuracy. The SHAP method was utilized to evaluate the contribution of features within the optimal predictive model, and the favorable clinical net benefit of the GBDT model was established through DCA. Given the uncertainties associated with PCNL, a clinical model that integrates multiple conventional parameters may prove to be more effective than relying on any single parameter (34, 35). To achieve this goal, we employed advanced machine learning techniques, which have been applied in preventing and managing stone treatment outcomes, to build predictive models based on common clinical parameters that are both simple and technically undemanding, making them suitable for use at the grassroots level. This approach enhances the potential for hospital implementation, significantly broadening the applicability of this study.

However, this study has several limitations. As a retrospective analysis, despite the implementation of stringent inclusion and exclusion criteria, there remains the potential for selection bias. The sample size was relatively small, which may limit the generalizability of the results, and the model was developed using data from a single center, potentially restricting its broader applicability. Importantly, the reliance on kidney-ureter-bladder (KUB) imaging for postoperative assessment introduces a significant limitation, as radiolucent stones (e.g., uric acid calculi) cannot be reliably detected. This not only affects the accuracy of residual stone evaluation but also confines the machine learning model’s training and validation to radiopaque stones, thereby limiting its generalizability across all stone types. Furthermore, the model was trained and tested exclusively on data from patients undergoing prone-position PCNL, and its performance may not extend to other surgical positions (e.g., supine or modified positions), which could influence stone localization, renal anatomy, and surgical outcomes. Future research should involve multi-center prospective studies incorporating a broader range of data, including diverse stone compositions, calyceal locations, and patient positioning, as well as more rigorous outcome assessments (e.g., advanced imaging modalities or standardized protocols). In addition, exploring advanced computational techniques, such as deep learning and artificial neural networks, may further enhance predictive accuracy and clinical utility.

## Conclusion

The prediction models based on GBDT, XGBoost, and RF showed strong effectiveness in predicting the stone-free rate after PCNL, with the GBDT model performing the best. It holds promise for aiding clinical decision-making and enabling personalized prevention and treatment strategies for patients with different residual stone risks.

## Data Availability

The raw data supporting the conclusions of this article will be made available by the authors, without undue reservation.

## Footnotes

^1^http://www.r-project.org/.

^2^https://www.python.org/downloads/release/python-380/.
